# Video Head Impulse Test: A Prognostic Marker for Patients with Idiopathic Sudden Sensorineural Hearing Loss

**DOI:** 10.3390/audiolres16010007

**Published:** 2025-12-31

**Authors:** Gaelle Ngankam Fotsing Epse Vofo, Matityahou Ormianer, Marrigje Aagje de Jong, Julia Meyler, Yaakov Noble, Ron Eliashar, Menachem Gross

**Affiliations:** 1 Department of Otolaryngology-Head & Neck Surgery, Hadassah-Hebrew University Medical Centre, Jerusalem 91120, Israel; matit@hadassah.org.il (M.O.); dejong.marije@gmail.com (M.A.d.J.); ronel@hadassah.org.il (R.E.); gross@hadassah.org.il (M.G.); 2 Shomea, Douala 5816, Cameroon; 3 Department of Language, Speech and Hearing Disorders, Hadassah-Hebrew University Medical Centre, Jerusalem 91120, Israel; jmeyler1954@gmail.com (J.M.); yaakovnoble@hadassah.org.il (Y.N.); 4 Faculty of Medicine, Hebrew University, Jerusalem 91120, Israel

**Keywords:** Idiopathic sudden sensorineural hearing loss, prognosis, recovery, dizziness, video head impulse test

## Abstract

**Background/Objectives**: Patients with Idiopathic sudden sensorineural hearing loss (ISSNHL) are often devastated by the unknown etiology coupled with the unknown pathway to recovery. The aim of this study was to evaluate whether abnormalities on the video Head Impulse Test (vHIT) performed early in the course of ISSNHL are associated with poorer hearing recovery. **Methods**: Forty-four patients with ISSNHL were prospectively enrolled between 2019 and 2022 following exclusion of differential diagnoses on clinical and MRI evaluation. vHIT was performed within 1–14 days of symptom onset and within 48 h of hospitalization. Recovery at six months was assessed both as a dichotomous outcome and by change in pure tone average (PTA). Group differences were analyzed using Fisher’s exact and Mann–Whitney U tests. A two-predictor logistic regression model examined the association between vHIT results, dizziness, and recovery. **Results**: Twelve patients exhibited abnormal vHIT findings. Abnormal vHIT was strongly associated with the presence of dizziness and with markedly poorer hearing recovery at six months. Patients with normal vHIT demonstrated substantially greater improvement in PTA thresholds compared with those showing abnormal results. Logistic regression further confirmed that abnormal vHIT was an independent predictor of reduced likelihood of hearing recovery, whereas dizziness alone did not independently influence outcomes. **Conclusions:** Our findings suggest that abnormal vHIT results in ISSNHL patients are linked to poor hearing recovery, which can enhance patient counseling regarding expectations. Although promising as a prognostic tool, we acknowledge our limited sample size and recommend validation in larger prospective cohorts.

## 1. Introduction

Idiopathic Sudden Sensorineural Hearing Loss (ISSNHL) is defined as sensorineural hearing loss of abrupt onset affecting at least 30 decibels (dB) in 3 contiguous frequencies for 3 days or less [1,2,3]. ISSNHL has an estimated incidence ranging between 5 and 20 per 100,000 persons per year [4], and as the name implies, its etiology is often unknown [1,5,6,7].

Although the auditory and vestibular systems are clearly distinct from one another, their mechanisms of neural transmission are identical. Many identifiable etiologies of hearing loss have also been associated with vestibular impairments [6,8]. Approximately 30–60% of patients with ISSNHL present with concomitant vestibular symptoms occurring at the onset of hearing loss or may be delayed for hours or even days [6,8,9,10]. Based on previous studies by Vofo et al., it was concluded that vestibular end organs were both subjectively and objectively affected in ISSNHL, as demonstrated by the abnormal electronystagmography (ENG) and caloric tests in 89% of patients with ISSNHL [11]. Concomitant vestibular involvement was also reported to carry a poorer prognosis [9,11].

Hamish MacDougall invented tight-fitting goggles with minimal slippage and a very clever way of mounting. It encompasses a small, lightweight, high-speed video camera in its goggles to provide a clear, sharp image of the eye during the head turn [12]. This technology is the video head impulse test (vHIT) technology. To date, the vHIT system has been used following a standard protocol using head impulses to test all six Semi-Circular Canals’ (SCC) function. It is also being used to test smooth pursuit and visual-vestibular interactions. vHIT is a physiological and rapid test, which studies the Vestibulo-Ocular Reflex (VOR) at high frequency for each SCC by calculating the duration ratio between the head impulse and gaze deviation. vHIT is more sensitive than the clinical head impulse test, especially in patients with isolated covert saccades. vHIT is a diagnostic test of vestibular weakness by gain reduction and the appearance of overt and covert saccades.

Saccades are fast, brief, accurate, and conjugate eye movements generated by the brainstem. They reposition the fovea onto the visual scene to maximize the visual acuity of specific regions of interest [13]. When the vestibular stimulus is short and abrupt, such as in small and rapid movements of the head, the VOR produces compensatory eye movements in the direction opposite to head movement. This aims to keep visual targets stable on the macula despite head movement. However, when the VOR is deficient, the brainstem triggers a rapid ocular movement in the same direction as the VOR to compensate for the insufficiency and reduce visual error [14]. This eye movement is called a “catch-up” saccade [15]. The vHIT quantitatively studies the VOR performance during a rapid head impulse imposed by the examiner [12]. The efficacy of VOR is defined either by the gain of the slow eye movement to the corresponding head movement or by the presence or absence of systematic corrective (or compensatory) catch-up saccades. Two types of catch-up saccades have been described: overt saccades and covert saccades. These differ in latency, and therefore, it is likely they have different mechanisms. Covert saccades have extremely short latency within the range of so-called “express saccades” (<100 ms) and occur during the head movement phase. On the other hand, overt saccades have latencies similar to visually guided saccades (150–250 ms) [16]. Covert saccades are probably triggered by vestibular signals, as vision is obscured during a head impulse, suggesting that they may be similar to fast phases. Overt saccades are usually made after the head has come to rest, and thus have a visual trigger, but might also be driven by vestibular signals. This test evaluates each SCC and helps in identifying isolated canal weaknesses.

The utility of vHIT in patients with ISSNHL is unclear and not established yet, specifically as a prognostic tool. A retrospective study described the use of vHIT in assessing only the posterior SCC in patients with ISSNHL but did not assess all the SCCs [17], while a study by Hepkarsi et al. performed both Caloric tests and vHIT to evaluate vestibular system involvement in patients with ISSNHL reporting concomitant involvement [18].

The purpose of this study was to determine the utility of vHIT in the diagnosis of subtle vestibular system impairment by evaluating all six SCCs in patients with ISSNHL and to evaluate its possible prognostic role.

## 2. Materials and Methods

A prospective observational (cohort) study involving patients with ISSNHL was performed. Helsinki approval was obtained from our Institutional Review Board), and patients signed a written informed consent form on admission to the Otolaryngology inpatient department. To determine the relationship between ISSNHL and vestibular impairment, all adult patients who were diagnosed with ISSNHL and treated at our tertiary University Medical Center were potential participants for the study.

**Inclusion criteria**: encompassed patients with unilateral ISSNHL, irrespective of the severity (based on pure tone average), with or without tinnitus or dizziness, of unknown origin, and no involvement of cranial nerves other than the eighth cranial nerve.

**Exclusion criteria**: patients with known causes of hearing loss (noise-induced, drug-induced, otitis media, herpes zoster oticus, trauma, vestibular migraine, Meniere’s disease, or other defined vestibulopathies), familial deafness, and chronic use of steroids or immunosuppressant therapy were excluded. Excluding vestibular migraine was particularly important given the recognized cerebrovascular association with SSNHL. Equally excluded were patients with onset of hearing loss lasting for more than one month without any attempted treatment.

Following the acquisition of written informed consent, patients were enrolled in the study. Demographic and clinical information were collected, including age, sex, side of hearing loss, vascular risk factors (hypertension, diabetes, smoking), and history of migraine. It is worth noting that patients who declined participation in the study were treated using the same protocol as study participants in accordance with our departmental protocol.

A clinical history and complete objective otolaryngology evaluation were obtained. Comprehensive audio-vestibular testing, including pure-tone audiometry (including 250 to 8000 Hertz (Hz) frequencies), tympanometry, speech perception threshold, and speech discrimination tests, caloric irrigation, and video Head Impulse Testing (vHIT) was performed. The vHIT evaluated all six semicircular canals (SCCs) with at least 15–20 head impulses per canal, to minimize gain variability and ensure statistical robustness. The vHIT was performed within 1–14 days of symptom onset and within 48 h of hospitalization. Follow-up hearing evaluations were conducted at 6 months.

The primary vestibular variables measured at baseline were Vestibulo-ocular reflex (VOR) gain and the presence or absence of overt and covert saccades for each SCC. VOR gains were automatically computed by the Integrated Clinical Solutions (ICS) Impulse system (GN Otometrics, ICS Impulse System, Model 1085, Taastrup, Denmark) and verified manually by two blinded investigators. Data was averaged across three artifact-free runs for each canal and reported to the physicians as normal or abnormal.

Imaging studies obtained included Magnetic Resonance Imaging (MRI) with a protocol consistent with Inner ear, Internal Auditory Canal, and Cerebellopontine Angle visualization to rule out acoustic neurinoma, inner ear malformations, and other pathological processes. The MRI equipment details vary since patients underwent scans at hospitals affiliated with their insurance scheme.

### 2.1. vHIT Test

Objective vestibular function tests by vHIT were performed in a darkened room within 48 h of admission to the department and between 1 and 14 days of symptom onset. No vestibular sedatives were administered prior to vestibular function testing. Eye movements were evaluated using a tight-fitting goggle and a video camera mounted on it, with the Eyeseecam program (Interacoustics, Middelfart, Denmark). VOR gains and saccades were evaluated. To classify vHIT results, we used a VOR gain cut-off of 0.8, widely applied in vestibular research and supported by published normative data. Alfarghal et al. and Patterson et al. showed that normal VOR gain values for healthy adults consistently cluster above 0.8 across the semicircular canals, suggesting that ≤0.8 is a practical boundary indicating potential vestibular hypofunction [19,20]. Similarly, Sinha et al. reported significantly reduced VOR gains in pathological groups when compared with normal-hearing controls, with abnormal ears typically falling below the 0.8 threshold in at least one canal [21]. Together, these studies provide empirical justification for applying 0.8 as a conservative threshold that balances sensitivity and specificity for detecting Vestibulo-ocular reflex impairment in clinical and research settings. Therefore, a VOR gain < 0.8 in any canal was considered abnormal in this study.

Of note, the vHIT operator was blind to the patients’ clinical data and audiometry test results.

### 2.2. Audiometric Parameters

The mean pure tone hearing threshold for 6 frequencies (250 to 8000 Hz) was analyzed for each patient upon admission. We feel that this mean value more accurately reflects the hearing status, as some patients have low-tone, high-tone, or flat hearing loss. By using only 3 or 4 frequencies, accurate evaluation of the hearing status may not be possible; for example, using only 3 frequencies, such as the pure tone average assessed at 500, 1000, and 2000 Hz, it is not possible to determine the real status of a patient with high-tone hearing loss and vice versa.

The Katz scale of degree of hearing loss was utilized with determinations of hearing loss scaled as: slight 16–25 dB hearing loss, mild 26–40 dB, moderate 41–55 dB, moderate to severe 56–70 dB, severe 71–90 dB, and profound > 90 dB hearing loss [22]. Repeat hearing assessment was performed at least 6 months following initiation of treatment according to the criteria proposed by Chang and colleagues in 2005 [23].

Hearing gains were determined as the parameter for hearing recovery as described by Siegel [24]; Hearing gain being an absolute value of hearing recovery, representing the average change in hearing levels from the initial to the final hearing test.

- Patients with no improvement were those who showed less than 15 dB gains in hearing.
- Patients with slight improvement were those who had more than 15 dB gain and a final hearing level poorer than 45 dB.
- Patients with partial recovery were those who gained more than 15 dB in hearing and had a final hearing level between 25 and 45 dB.
- Patients with complete recovery were those with a final hearing level better than 25 dB, regardless of the amount of gain.

For the purpose of statistical analysis, hearing recovery was also dichotomized as 1 for partial or complete recovery, or 2 for slight or no recovery.

Treatment was initiated as early as possible with oral corticosteroids (prednisone) at a dose of one milligram (mg) per kilogram per day, with the usual maximum dose of 60 mg daily prescribed for 7 to 14 days, then tapered over a similar period for all patients following the diagnosis of ISSNHL. If no improvement was detected, hyperbaric oxygen therapy was added, and intra-tympanic steroid injections were initiated as salvage therapy. This is in accordance with the American Academy of Otolaryngology-Head and Neck Surgery Clinical Practice Guidelines [25].

### 2.3. Statistical Analysis

Statistical analysis was performed using SPSS version 25.0 (IBM Corp., Armonk, NY, USA). Continuous variables were tested for normality and summarized as mean ± standard deviation (SD) or median with range where appropriate. Categorical variables were summarized as frequencies and percentages.

Group comparisons for categorical outcomes were performed using two-tailed Fisher’s exact tests, with odds ratios (OR) and 95% confidence intervals (CI) reported. Continuous variables that were non-normally distributed were compared using the Mann–Whitney U test, with effect sizes reported as rank-biserial correlation.

To evaluate factors associated with recovery at 6 months, a binary logistic regression model was applied with hearing recovery (yes/no) as the dependent variable. Due to limited outcome events, only two predictors (vHIT result and presence of dizziness) were included to reduce overfitting. Regression results are reported using β coefficients, standard errors, OR, 95% CI, and *p*-values.

All statistical tests were two-tailed, and significance was set at *p* ≤ 0.05. Patients who did not attend the 6-month follow-up were excluded from recovery outcome analyses.

## 3. Results

### 3.1. Study Population

Forty-four patients with ISSNHL were included between 2019 and 2022. After exclusion of eight patients with alternative diagnoses (Meniere’s disease, vestibular migraine, acoustic neuroma, demyelinating disease), the final cohort had a mean age of 41.8 ± 16.6 years (18–77 years), and 24 (54.5%) were female. Median time from symptom onset to presentation was 3 days (range 1–31). Mean time from symptom onset to vHIT testing was 4.6 ± 3.1 days (range 1–14), which may act as a confounder.

Associated tinnitus was present in 34/44 (77.3%) patients, and dizziness in 18/44 (40.9%) patients with ISSNHL in our cohort.

Treatment was initiated upon admission, and 29/44 (65.9%) received systemic steroids alone, 6/44 (13.6%) received intratympanic steroids alone, while 9/44 (20.5%) received combined therapy with salvage intratympanic steroids usually following failure of systemic steroids. All patients who received combined therapy were also referred to hyperbaric oxygen therapy, which varied between 15 and 25 sessions. The presence of tinnitus and treatment modality (systemic and/or intratympanic steroids with or without hyperbaric oxygen therapy) did not show any significance to the hearing recovery. Table 1 summarizes the above-mentioned characteristics.

### 3.2. vHIT Findings

vHIT results were classified as abnormal based on VOR gain <0.8 in the stimulated canal and/or presence of consistent overt or covert corrective saccades.

Twelve patients (27.3%) had abnormal vHIT (Figure S2 vs. Figure S1, the latter showing a normal vHIT), with posterior canal involvement most frequent (59%), followed by lateral (29%) and superior (12%). All six SCCs were assessed in each patient. We summarized abnormal results as abnormal irrespective of the SCCs involved. All abnormal results corresponded to the side of the patient affected by ISSNHL.

Patients with ISSNHL were stratified into four groups:

- Dizziness and positive (abnormal) VHIT (Dizziness+/vHIT+) with 10 patients (22.8%, *n* = 44)
- Dizziness and negative (normal) VHIT (Dizziness+/vHIT−) with 8 patients (18.2%, *n* = 44)
- No dizziness and positive (abnormal) VHIT (Dizziness−/vHIT+) with 2 patients (4.5%, *n* = 44)
- No dizziness and negative (normal) VHIT (Dizziness−/vHIT−) with 24 patients (54.5%, *n* = 44)

The proportion of abnormal vHIT differed sharply between groups: in patients with dizziness, 10 out of 18 (55.6%) had abnormal vHIT results compared to 2 out of 26 (7.7%) amongst patients without dizziness. This difference was statistically significant (Fisher’s Exact Test, *p* = 0.002, OR 13.6, 95% CI 2.4–92.1).

### 3.3. Recovery Outcomes

Six-month hearing recovery was assessed both as a dichotomous outcome (recovered vs. not recovered) and a change in pure tone average (PTA) in decibels.

### 3.4. Group Outcomes (N = 44)

#### Comparisons

When hearing recovery was examined across the four predefined subgroups (dizziness+/vHIT+, dizziness+/vHIT−, dizziness−/vHIT+, dizziness−/vHIT−), clear patterns emerged. Patients with normal vHIT findings showed a more favorable auditory improvement over six months, whereas those with abnormal vHIT tended to have more limited recovery. This is shown in Figure S3, a box plot that illustrates the distributions of baseline and six-month PTA thresholds by vestibular status. After Bonferroni correction, differences remained most pronounced between patients with dizziness plus abnormal vHIT and those with normal vestibular responses.

- Dizziness+/vHIT+ vs. Dizziness+/vHIT−
  Fisher’s Exact Test: *p* = 0.04, OR 6.7 (95% CI 1.02–49.8)
- Dizziness+/vHIT+ vs. Dizziness−/vHIT−
  Fisher’s Exact Test: *p* = 0.019, OR 9.1 (95% CI 1.4–60.3)

The mean PTA improvement at 6 months was +23.4 ± 11.1 dB in patients with normal vHIT and +9.5 ± 7.8 dB in patients with abnormal vHIT. Mann–Whitney U: *p* = 0.006

### 3.5. Predictive Values

Base rate of recovery in the entire cohort was 54.5% (24/44). Our negative vHIT predicting no recovery (NPV) was 91.7% (95% CI 61.5–99.8); negative vHIT predicting recovery (PPV) was 68.4% (95% CI 46.1–85.6). On the other hand, our absence of dizziness predicting recovery (PPV) was 69.2% (95% CI 48.9–85.6).

### 3.6. Multivariate Regression

Due to the limited number of outcome events, only two predictors were included to avoid overfitting: abnormal vHIT result (OR 4.98; β = 1.60, SE = 0.72. 95% CI 1.17–23.2 *p* = 0.029) and the presence of dizziness (OR 2.04; β = 0.71, SE = 0.64. 95% CI 0.58–8.2 *p* = 0.15) as shown in Figure S4 which summarizes the independent predictors of hearing recovery using logistic regression. The model explained Nagelkerke R^2^ = 0.24 of the variance in recovery. However, in the logistic regression model adjusting for age, sex, and baseline PTA, abnormal vHIT remained an independent predictor of poorer hearing recovery at six months. In contrast, dizziness alone did not independently predict outcome once vestibular test results and baseline hearing were considered. These findings support the role of early vHIT evaluation as a clinically relevant prognostic tool.

## 4. Discussion

The findings from our study on the predictive capacity of video Head Impulse test (vHIT) in patients with Idiopathic Sudden Sensorineural Hearing Loss (ISSNHL) shed light on the nuanced interplay between vestibular function and auditory outcomes. We support the growing evidence that vHIT provides a valuable, non-invasive tool for assessing vestibular integrity in ISSNHL. Specifically, early vHIT abnormalities appear to be an independent negative prognostic indicator for long-term hearing recovery, suggesting concomitant labyrinthine or neural dysfunction [4,5,6,10,11]. This study’s novelty lies not only in the demonstration of vHIT as a prognostic tool but also in the comprehensive patient profiling and analysis of associated clinical symptoms—insights that could reshape routine clinical practices for managing ISSNHL.

A prospective cohort study was carried out in patients admitted with ISSNHL to evaluate the association between dizziness, positive findings on vHIT, and hearing recovery rate. After excluding those with other vestibular pathologies like Vestibular Migraine, Meniere’s disease, and abnormal MRI findings, our cohort of 44 patients with a mean age of 41.8 years reflects a demographic that is both diverse and significant, as ISSNHL is known to affect individuals across various age groups. Of note, over fifty percent of our cohort were female. Studies by Rauch et al. reported similar age groups but stated an equal gender distribution [6]. Our study shows that 40.9% of the patients reported hearing impairment associated with dizziness, which corroborates previous literature and highlights the complex symptomatology that often accompanies ISSNHL [6], supporting the known fact that auditory impairment can occur in association with vestibular disturbances [4,5,6,9,10,11].

All patients had vHIT performed amongst other tests, and it was shown that most of the patients with dizziness had abnormal vHIT results when compared to patients without dizziness. Dizziness is a known indicator of vestibular system involvement when other neurological causes have been excluded. Our study supports the fact that, even though this symptom can be of variable severity, subtle changes can be detected on vHIT, a fact equally reported by Byun et al. [17].

Nakamichi et al. reported that the SCC affected varied with the vestibular pathology when comparing patients with SSNHL with dizziness and patients with Vestibular neuritis [26], but our study included only patients with ISSNHL, irrespective of the affected SCC.

By stratifying patients based on the presence of dizziness and the results of vHIT, we provided a clear framework for understanding the implications of vestibular assessments in prognostic outcomes. Our analysis demonstrated a compelling correlation between vHIT outcomes and hearing recovery, as patient recovery was worse in patients with Dizziness+/vHIT+ when compared to patients with Dizziness+/vHIT− and Dizziness−/vHIT−. The significant differences in recovery rates among the four patient groups affirm the diagnostic relevance of the vHIT: those demonstrating both dizziness and abnormal vHIT were less likely to recover hearing, illustrating not just a dual symptom presentation but a potential compounded risk for poor auditory outcomes.

Moreover, the statistical significance of vHIT results at baseline in our regression model emphasizes the importance of conducting vHIT assessments early in the course of treatment. The established predictive values (PPV of 68.4 and NPV of 91.7) position vHIT as a pivotal component of the prognostic evaluation toolkit for clinicians. This ability to predict outcomes based on early vestibular assessments presents a strong case for integrating vHIT into standard practice for patients presenting with ISSNHL. Byun et al. reported poorer prognosis in ISSNHL patients with abnormal posterior SCC function [17], but they did not evaluate or predict recovery based on a six-semicircular-canal-test result like the vHIT. Although previous studies have suggested the reliability of caloric testing (which examines low-frequency vestibular responsiveness) as a prognostic marker, our data indicate that vHIT abnormalities (evaluating high-frequency vestibular function), particularly reduced VOR gain and overt saccades, may reflect acute utricular and semicircular canal involvement, offering earlier insight into the extent of labyrinthine injury [18]. Conversely, another study did not find any statistically significant difference in vestibular function using vHIT between patients with ISSNHL with and without dizziness, probably due to their smaller sample size of thirty patients [27].

The timing of treatment onset was assessed and reported to be mostly within 3 days of the onset of symptoms. Patient recovery based on this parameter was not found to be statistically significant. This is in line with what was reported previously [28].

Given our modest sample size and observational design, we believe it would be inappropriate to draw firm conclusions or make treatment recommendations such as higher systemic steroid dosages, routine primary intratympanic steroid injections, or early hyperbaric oxygen therapy solely on the basis of vHIT abnormalities. Such inferences would require larger, preferably multicenter, prospective studies specifically designed to assess whether vestibular involvement modifies treatment response. Nonetheless, we agree that our findings have important clinical relevance. Identification of vestibular deficits at presentation may help clinicians better stratify prognosis and provide more accurate counseling regarding expected hearing recovery. Patients with abnormal vHIT may benefit from closer follow-up and heightened clinical vigilance. Future studies with larger cohorts should explore whether early treatment intensification in this subgroup translates into improved outcomes.

Hearing severity was neither affected by the presence of dizziness nor by the presence of abnormal vHIT in our study population, stating that the magnitude of neural involvement in both vestibular and cochlear nerves is different. This is contrary to what was reported by Niu and colleagues, who found that patients with SSNHL who presented with dizziness or abnormal caloric test displayed worse hearing loss; and vice versa, and that dizziness and abnormal caloric results happened more frequently in SSNHL patients with profound hearing loss. This difference could be explained by their larger sample size of 149 patients [26].

In multivariate analysis, our study reinforces the notion that traditional factors such as age, gender, laterality, or duration of symptoms may not exert the anticipated influence on recovery rates, shifting the focus instead to the functional impairments indicated by vHIT. The lack of significance for these parameters urges a re-evaluation of current prognostic models that often rely heavily on demographic and clinical histories. Additionally, the presence of tinnitus and treatment modality (systemic and/or intratympanic steroids with or without hyperbaric oxygen therapy) did not show any significance, but we acknowledge our small sample size and a relatively young population.

As we advocate for the routine utilization of vHIT in ISSNHL patients, we acknowledge the potential for this tool not only to tailor treatment strategies but also to enhance patient counseling regarding likely outcomes. The high NPV suggests a reliable safety net for identifying patients with a better prognosis, enabling healthcare providers to make more informed decisions regarding follow-up interventions and resources.

### 4.1. Relationship Between Dizziness and vHIT

While dizziness alone was associated with poorer outcomes, vHIT provided greater discriminatory value. Many patients with clinical dizziness had normal vestibular testing, supporting the notion that symptoms and measurable impairment do not always align.

### 4.2. Study Limitations

Some limitations of our study are our relatively small sample size and the absence of a control group. However, the canal-specific analysis and the use of standardized vHIT protocols strengthen the internal validity of the findings. A further limitation of this study is the absence of repeat vHIT assessments at the six-month follow-up. In the present design, vestibular function was evaluated only at baseline, as the primary objective was to investigate the prognostic value of early vestibular involvement on subsequent hearing recovery. Hearing outcomes were therefore monitored longitudinally, while vestibular testing was not repeated.

We acknowledge that vestibulo-ocular reflex (VOR) gains measured by vHIT may evolve over time due to vestibular compensation, and follow-up testing could have provided valuable insights into the temporal relationship between vestibular recovery and auditory outcomes. However, incorporating serial vHIT assessments was beyond the scope of this study and was limited by real-world clinical follow-up constraints.

Future prospective studies with systematic longitudinal vestibular testing are warranted to better characterize vestibular recovery patterns and to determine whether changes in vHIT parameters over time correlate with hearing recovery or long-term functional outcomes.

### 4.3. Timing as a Confounder

A key methodological limitation is variability in time between symptom onset and vHIT testing (1–14 days). Neural or end-organ recovery may already have begun in some patients by the time of testing. A sensitivity analysis adjusting for onset-to-testing delay did not materially change group trends, but future research should standardize the testing window.

### 4.4. MRI Protocol Variability

Exclusion of retro-cochlear pathology relied on MRI reports from different scanners and institutions. Variation in resolution and sequences could affect diagnostic consistency, and we acknowledge this as a limitation.

### 4.5. Statistical Limitations

Only 12 patients had abnormal vHIT, which:

- Limits statistical power
- Violates conventional 10-events-per-variable rules for multivariate regression
- Increases risk of overfitting

We addressed this by restricting the regression model to two predictors and emphasizing that the results are exploratory rather than confirmatory.

### 4.6. Recovery Measurement

Reporting both dichotomous recovery and continuous PTA shift provides more clinical nuance. However, future studies should explore model-based prediction using continuous outcomes (e.g., ordinal modeling).

### 4.7. Need for Larger and Prospective Validation

The findings require cautious interpretation and prospective multicenter validation with:

- Larger sample size
- Standardized vestibular test protocols
- Uniform MRI acquisition
- Stratified analysis based on the timing of assessment

## 5. Conclusions

In summary, our study demonstrates that early identification of vHIT abnormalities, particularly low VOR gains and the presence of saccades, is associated with poorer hearing recovery following ISSNHL and smaller improvement in PTA at 6 months. While vHIT holds promise as a rapid, non-invasive prognostic tool, the present study should be regarded as preliminary due to its limited sample size and potential confounders. Larger, standardized studies are needed to confirm optimal prognostic thresholds and establish vHIT as a routine element of ISSNHL assessment.

## Figures and Tables

**Table 1:** Baseline Characteristics of Participants.

Baseline Characteristic	Dizziness+ VHIT+		Dizziness+ VHIT−		Dizziness− VHIT+		Dizziness− vHIT−		Full Sample	
	*n* = 10	%	*n* = 8	%	*n* = 2	%	*n* = 24	%	*N* = 44	%
Gender										
Female	7	70.0	7	87.5	0	50.0	10	41.7	24	54.5
Male	3	30.0	1	12.5	2	50.0	14	58.3	20	45.5
Age (Years)										
Mean	48.2		37.4		41.0		40.0		41.8	N/A
Standard Deviation	16.98		17.07		32.53		15.62		16.62	N/A
Median	52.5		36		41		40.5		42	N/A
Range	19–68		18–68		18, 64		20–77		18–77	N/A
Duration of symptoms (days)										
Mean	5.1		7.3		11		6.3		7.4	N/A
Standard Deviation	3.93		10.02		2.83		7.94		7.47	N/A
Median	4.5		3		11		3		3	N/A
Range	1–14		1–32		14, 32		1–32		1–32	N/A
Hearing level at enrolment										
Mild	2	20.0	2	25.0	1	50.0	3	12.5	8	18.2
Moderate	1	10.0	0	0	0	0	10	41.6	11	25.0
Severe	2	20.0	4	50.0	1	50.0	7	29.2	14	31.8
Profound	5	50.0	2	25.0	0	0	4	16.7	11	25.0
Dizziness	10	100	8	100	0	0	0	0	18	40.9
Tinnitus	9	90.0	8	100	2	100	15	62.5	34	77.3
Treatment received										
Systemic steroids only	3	30.0	5	62.5	2	100	19	79.2	29	64.9
Intratympanic steroids only	1	10.0	0	0	0	0	1	4.2	2	4.5
Both systemic and intratympanic steroids	6	60.0	3	37.5	0	0	4	16.6	13	29.6

*N* = 44 (*n* = number for each characteristic). N/A = Not Applicable.

## Data Availability

The data presented in this study are available on request from the corresponding author due to privacy and ethical restrictions. All data and materials support our published claims and comply with field standards.

## Supplementary Materials

The following supporting information can be downloaded at: https://www.mdpi.com/article/10.3390/audiolres16010007/s1, Figure S1: Normal video head impulse test results in a patient with idiopathic sudden sensorineural hearing loss. Figure S2: Abnormal video head impulse test result in a patient with idiopathic sudden sensorineural hearing loss. Figure S3: Changes in pure tone average (PTA) among patients with dizziness and abnormal vHIT. Figure S4: Forest plot of predictors of hearing recovery at six months.
