# Supplementary material for: Video Head Impulse Test: A Prognostic Marker for Patients with Idiopathic Sudden Sensorineural Hearing Loss
Source: Audiol Res. 2025 Dec 31;16(1):7. doi: 10.3390/audiolres16010007 (PMC12821494; doi:10.3390/audiolres16010007)
Supplement: Supplementary file 1 [file audiolres-16-00007-s001.zip › Figure S2.pdf]

FIGURE S2

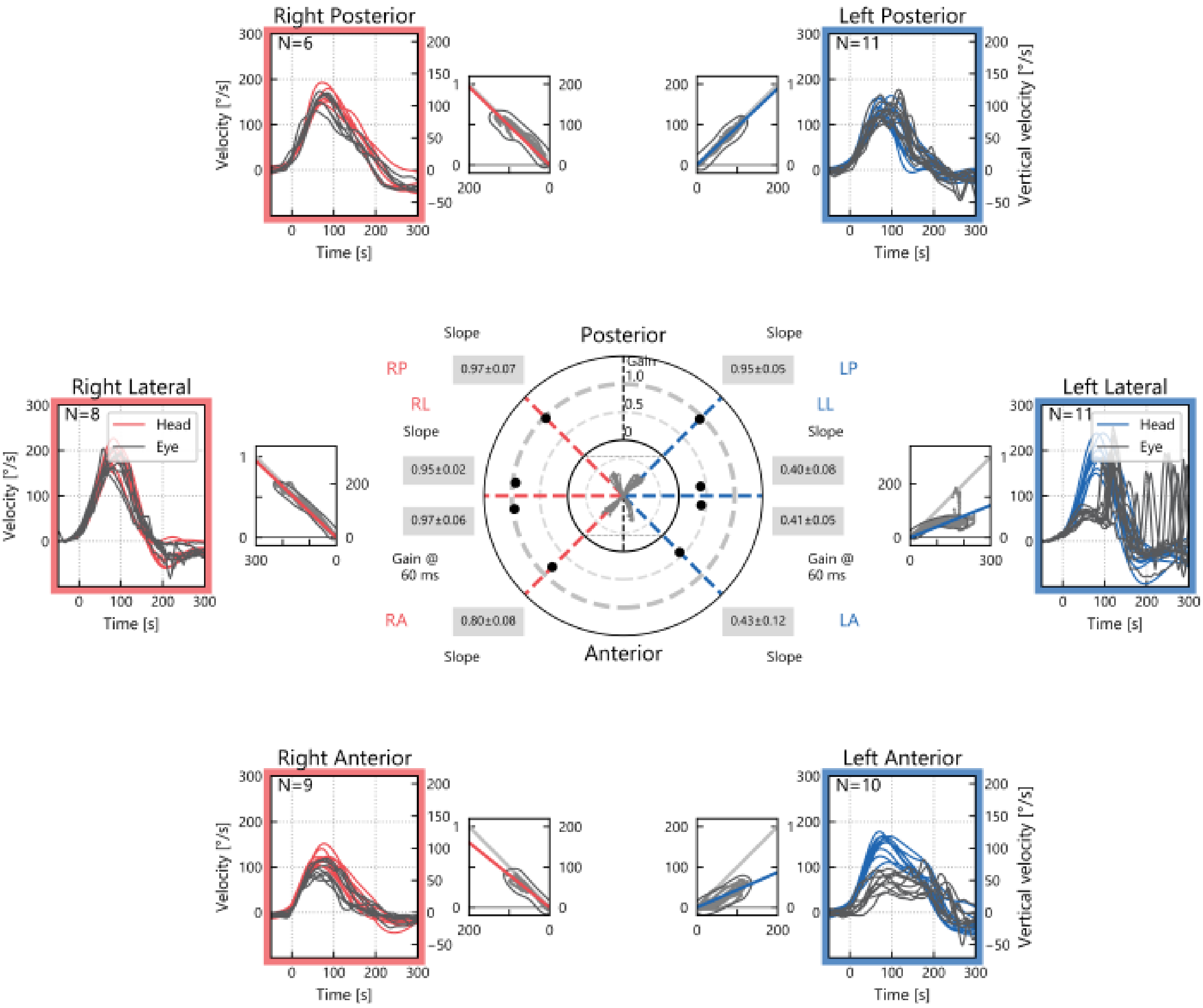

Figure S2:

Title: Abnormal video head impulse test result in a patient with idiopathic sudden sensorineural hearing loss.

Legend:

Video head impulse testing of all six semi-circular canals showing abnormal results in a 29-year-old male with idiopathic sudden sensorineural hearing loss with dizziness. There are abnormal catch-up saccades and VOR gains on the left lateral semi-circular canal.
