# Supplementary material for: Video Head Impulse Test: A Prognostic Marker for Patients with Idiopathic Sudden Sensorineural Hearing Loss
Source: Audiol Res. 2025 Dec 31;16(1):7. doi: 10.3390/audiolres16010007 (PMC12821494; doi:10.3390/audiolres16010007)
Supplement: Supplementary file 1 [file audiolres-16-00007-s001.zip › Figure S3.pdf]

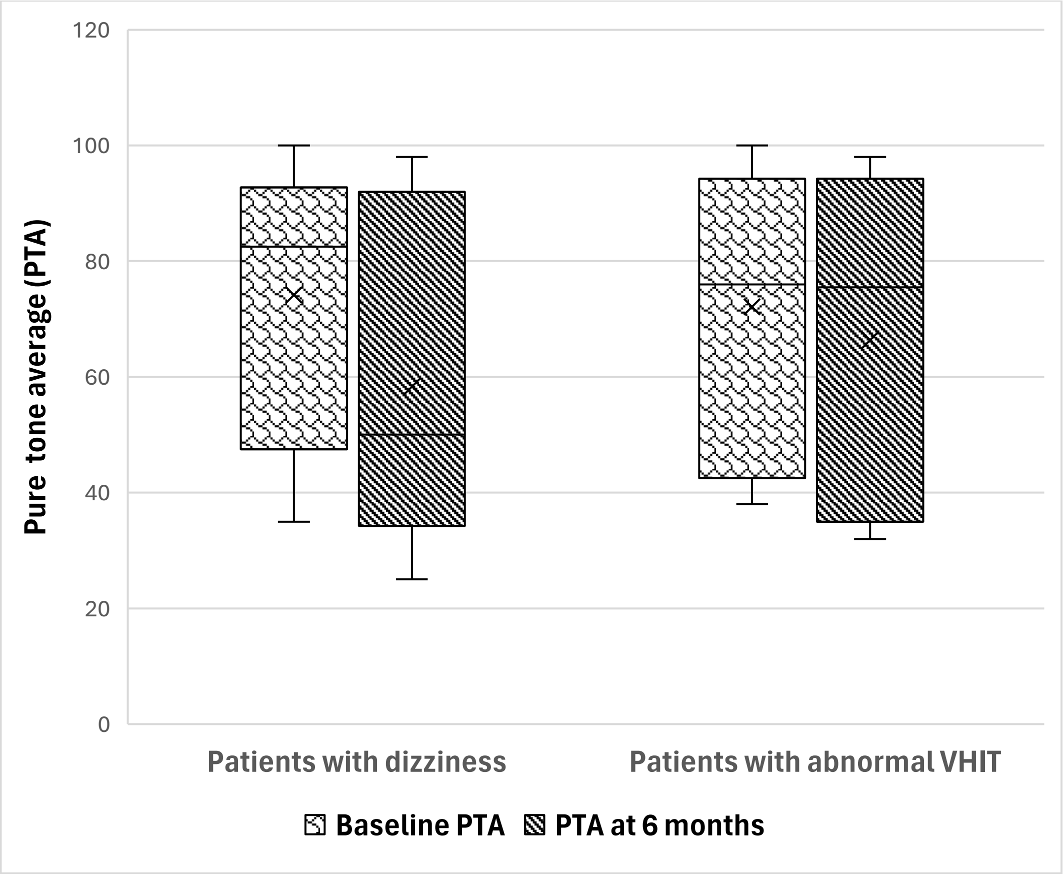

Figure S3: Changes in pure tone average (PTA) among patients with dizziness and abnormal vHIT
Box plots comparing baseline and six-month pure tone average (PTA) thresholds among patients with dizziness and those with abnormal vHIT. Boxes represent the interquartile range (IQR), the horizontal line indicates the median, and whiskers denote the minimum and maximum values.
