# Supplementary material for: Video Head Impulse Test: A Prognostic Marker for Patients with Idiopathic Sudden Sensorineural Hearing Loss
Source: Audiol Res. 2025 Dec 31;16(1):7. doi: 10.3390/audiolres16010007 (PMC12821494; doi:10.3390/audiolres16010007)
Supplement: Supplementary file 1 [file audiolres-16-00007-s001.zip › Figure S4.pdf]

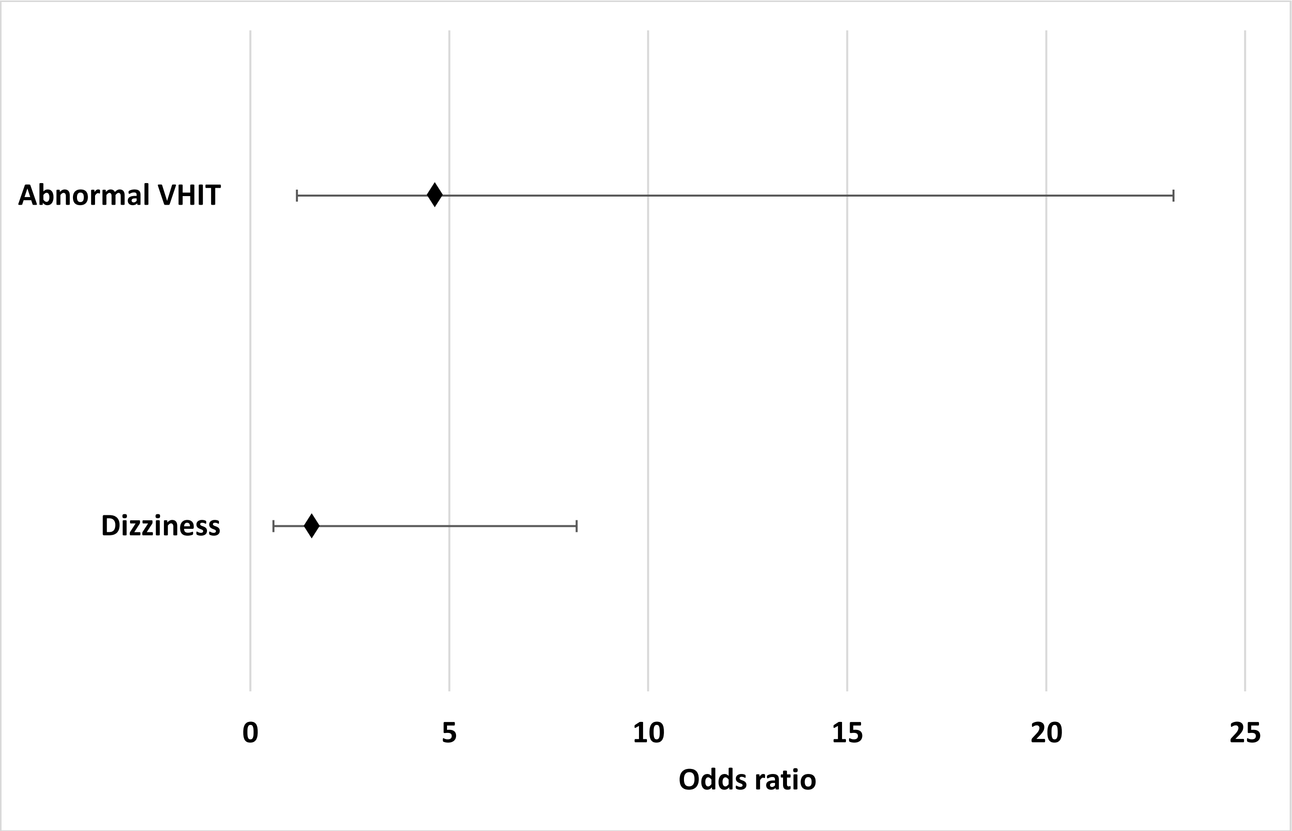

Figure S4: Forest plot of predictors of hearing recovery at six months

Forest plot illustrating the odds ratios (ORs) and 95% confidence intervals for predictors of hearing recovery at six months following idiopathic sudden sensorineural hearing loss. Abnormal vHIT was associated with reduced odds of hearing recovery, whereas dizziness alone showed a weaker and non-significant association. The horizontal lines represent 95% confidence intervals, and the vertical reference line indicates an odds ratio of 1.0.
